# Invariant based quartet puzzling

**DOI:** 10.1186/1748-7188-7-35

**Published:** 2012-12-06

**Authors:** Joseph P Rusinko, Brian Hipp

**Affiliations:** 1 Department of Mathematics, Winthrop University, 142 Bancroft Hall, Rock Hill, SC 29733, USA; 2 Department of Mathematics, Winthrop University, 142 Bancroft Hall, Rock Hill, SC 29733, USA

**Keywords:** Phylogenetic reconstruction, Invariants, Quartet puzzling

## Abstract

**Background:**

First proposed by Cavender and Felsenstein, and Lake, invariant based algorithms for phylogenetic reconstruction were widely dismissed by practicing biologists because invariants were perceived to have limited accuracy in constructing trees based on DNA sequences of reasonable length. Recent developments by algebraic geometers have led to the construction of lists of invariants which have been demonstrated to be more accurate on small sequences, but were limited in that they could only be used for trees with small numbers of taxa. We have developed and tested an invariant based quartet puzzling algorithm which is accurate and efficient for biologically reasonable data sets.

**Results:**

We found that our algorithm outperforms Maximum Likelihood based quartet puzzling on data sets simulated with low to medium evolutionary rates. For faster rates of evolution, invariant based quartet puzzling is reasonable but less effective than maximum likelihood based puzzling.

**Conclusions:**

This is a proof of concept algorithm which is not intended to replace existing reconstruction algorithms. Rather, the conclusion is that when seeking solutions to a new wave of phylogenetic problems (super tree algorithms, gene vs. species tree, mixture models), invariant based methods should be considered. This article demonstrates that invariants are a practical, reasonable and flexible source for reconstruction techniques.

## Background

### History

A phylogenetic tree provides a visual representation of the relationships among a collection of organisms. Accurate and easily computable phylogenies allow scientists to make informed decisions based on the genetic relationships among taxa. Phylogenies can then be used to fight disease outbreaks [1], to develop plans for saving endangered species [2], or to assemble the Tree of Life [3].

The majority of existing algorithms for phylogenetic reconstruction fall into one of three classes: distance based algorithms, parsimony algorithms, and maximum likelihood based algorithms. These classes of algorithms justifiably form the pillars of phylogenetic reconstruction, but they are each known to have shortcomings. Parsimony algorithms have difficulty in reconstructions involving rapidly evolving taxa. Maximum likelihood algorithms are typically slow and suffer from long branch attraction. Distance based algorithms also suffer from long branch attraction, and have regions where they are uncomputable due to infinite or negative distances.

In 1987 Cavender and Felsenstein [4], Lake [5], and Evans and Speed [6] introduced a new class of reconstruction algorithms based on *invariants*. These invariants are relationships which observed data should satisfy assuming the taxa have evolved over a given tree topology and model of evolution. Initial studies in invariant based reconstruction found it to be less effective than more traditional methods [7,8]. Due to their limited accuracy on sequences of a biologically reasonable length, invariant based algorithms fell out of favor in practical phylogenetics research. Upon closer inspection, the limited success of invariants is not surprising given that initial attempts did not use all possible expected relationships among the observed data. In this article, we build on mathematical advances in the field of algebraic geometry that have made it possible to reconsider invariants as a practical and flexible source for reconstruction algorithms [9-11].

### Application of algebraic geometry to phylogenetics

Recent work by algebraic geometers has led to the construction of complete lists of invariants for certain models of evolution [9,12,13]. Casanellas and Fernández-Sánchez used a complete list of invariants to analyze the performance of invariant based reconstruction of quartet trees and found that they performed quite well, and in some instances outperformed traditional methods such as neighbor joining and maximum likelihood [11]. Still the direct use of invariants on larger data sets would require the construction of a potentially enormous list of polynomial equations (determined by the tree topology), which must then be evaluated on each of the possible evolutionary trees. The computational power required to complete these tasks makes the direct use of invariants impractical for large data sets. As such, the resurgence of interest in invariant based phylogenies has been focused both around the development of new and exciting mathematics, and as a theoretical framework for proving that certain types of phylogenetic reconstructions are possible [9,14,15].

As a theoretical framework for understanding phylogenetics, invariant based methods are quite powerful. Unlike distance based methods, they can account for any conceivable evolutionary rate, and any number of differences per site. They have been used, for example, to demonstrate when it is possible to determine if a mixture model can be inferred from a data set [14]. However, practicing biologists have widely dismissed invariant based algorithms because there has not been a clear algorithm for applying them to realistic phylogenetic reconstruction problems.

### Invariant based quartet puzzling

To address the scalability issue, we propose a variation of quartet puzzling which uses invariants to compute the individual quartets, thus allowing the application of invariants to data sets of arbitrary size. Strimmer and von Haeseler introduced quartet puzzling as a way to take advantage of the theoretical power of maximum likelihood while limiting the computational costs involved in a full maximum likelihood reconstruction [16]. Subsequently, the use of quartet puzzling has become standard through programs such as TREE-PUZZLE [17]. Quartet puzzling computes the optimal four taxa trees for every subset of four taxa from the data set. A puzzling algorithm is used to combine these quartet trees into a large tree containing all of the taxa. Simulation studies of quartet puzzling revealed that errors made in the choice of the individual quartets were propagated throughout the puzzling process, which in some instances caused inaccurate tree reconstructions [18]. Attempts to reduce these errors include variations on quartet puzzling which limit the quartets that are examined, provide different weights for individual quartets, or modify the puzzling procedure [18-20]. Although it was conjectured that quartet based methods of reconstruction could not compete with neighbor joining algorithms for accuracy [18], the short quartet puzzling method of Snir et. al. disproved this claim by outperforming the neighbor joining algorithm [19].

In this paper we develop an analog of the original method proposed in [16]. Instead of using maximum likelihood to reconstruct the quartet trees, we use invariant based reconstruction. Our invariant reconstruction method follows [11] with the following modifications. We tested our algorithm using a modification of the algebraic Jukes-Cantor invariants for an unrooted quartet tree without molecular clock restrictions which was constructed in [9] and is available in [13]. We also used the algebraic Kimura 2-parameter invariants available on the same site. Using the metric outlined in [11], we select the quartet for which the sum of the absolute value of the evaluation of the invariants at the observed pattern frequencies is the smallest. We test the performance of this algorithm using simulated DNA sequence data.

## Methods

### Construction of invariants

**Figure 1:**
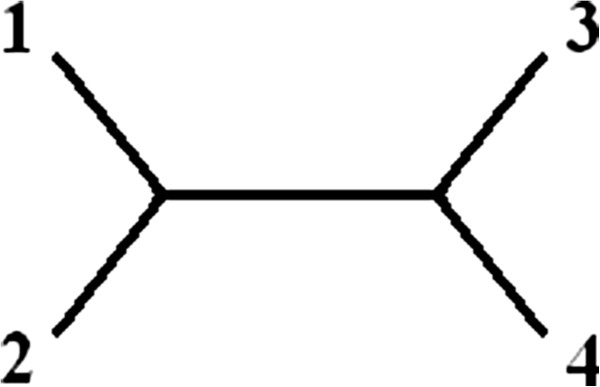
Depiction of Unrooted Tree Topology (12)(34).

If taxa have evolved under a particular evolutionary model along a given tree topology, the pattern frequencies which occur in the aligned nucleotide sequences should satisfy a set of polynomial relationships called invariants. For our study we test both the Jukes-Cantor, and the Kimura-2 parameter models of evolution for the unrooted quartet tree topology (12)(34) (see Figure 1). For each model, one can group pattern frequencies into classes *p*_1_,*p*_2_,⋯*p*_*n*_ for which the expected pattern frequencies are the same (see [13] for an explicit description).

In the Jukes Cantor model, two of the invariants which arise in the *p*_*i*_ s are linear invariants, called Lake’s invariants [5]. Each of these invariants can be expressed as a sum of four terms which is expected to equal zero. Sturmfels and Sullivant [9] found that the remaining invariants are much easier to compute after a linear change of coordinates based on a discrete Fourier transformation. This method was first applied in a phylogenetic setting by Evans and Speed [6]. After the change of coordinates, they label the new frequency classes *q*_1_,⋯,*q*_*n*_. In the *q*_*i*_ s, there are 33 additional invariants which can be expressed as binomial expressions (10 quadratic and 23 cubic) which are expected to equal zero when evaluated using the pattern frequencies for the Jukes Cantor Model. A similar construction for the Kimura-2 parameter model yields 795 expressions (54 quadratic, 390 cubic, and 351 quartic).

Given a collection of invariants, Casanellas and Fernández-Sánchez used the sum of the absolute values of the evaluation of each invariant expression as a score for how well a particular tree fits the data, and they selected the tree with the lowest score for phylogenetic reconstruction. They found this method to be quite accurate in selecting the best quartet arrangement [11]. While several scoring algorithms have been explored in the literature [10], we adopt Casanellas and Fernández-Sánchez’ metric because of its simplicity and accuracy. In this article we will also explore several additional collections of invariants used to reconstruct the quartet phylogeny.

### Relevant invariants

Recent work by Casanellas and Fernández-Sánchez, based on the geometry of the invariants and the combinatorics of binary trees, demonstrated that it is theoretically possible to reconstruct phylogenetic trees using a subset of the invariants called *relevant* invariants [21]. For the Jukes-Cantor model, they show that only the linear and quadratic invariants are *relevant*. The potential to use fewer invariants is appealing both in terms of algorithm speed and simplicity. In our study we seek to determine whether reconstruction accuracy is improved by evaluating the data using only the relevant invariants.

### Biologically symmetric invariants

The minimal collection of invariants used by Casanellas and Fernández-Sánchez arises from a statistical framework, but because they were derived using algebraic geometry, this particular list has one drawback. Biologically speaking we would like the trees (12)(34) and (21)(34) to have the same score since they represent the exact same relationship among the data. This is not the case, however, because the invariant expressions are not symmetric. The fact that two trees that represent the same relationship among the taxa can have different scores when evaluated at the observed pattern frequencies may cause serious theoretical problems for phylogenetic inference.

To illustrate the theoretical difficulties involved with using this minimal set of invariants we use an example with data drawn from the crab.meg file that comes with the MEGA software package [22]. We computed the minimal invariant scores for each of the 24 possible orderings of the taxa 1=Artemia_salina, 2=Clibanarius_vittatus, 3=Paralithodes_camtschatica and 4=Pagurus_acadianus. Table 1 lists the top ten scores among these orderings. Notice that while the best overall score corresponds to the pairing (13)(24) , the following three best scores all point to (14)(23) as the proper unrooted tree.

**Table 1:** Minimal invariant scores for crab data

**Ranking**	**Ordering**	**Score**	**Unrooted tree**
1	4231	.00660042	(13)(24)
2	2341	.00662293	(14)(23)
3	3214	.00664202	(14)(23)
4	3241	.00664335	(14)(23)
5	3142	.00668515	(13)(24)
6	3412	.00669565	(12)(34)
7	1234	.00672918	(12)(34)
8	4123	.00681494	(14)(23)
9	1432	.00683403	(14)(23)
10	4132	.00683536	(13)(23)

Minimal invariant scores for selected orderings of a four species subset of crab data.

Potential theoretical solutions to this problem have been explored by Erickson using algebraic geometry [10], and by Sumner et al. using representation theory [23,24]. We adopt Erickson’s solution which we briefly review here. The zero set of the collection of invariants describes a particular shape known as an algebraic variety. There are many different collections of equations whose zero sets describe the same geometric shape. The set of invariants computed in [13] is a minimal set of equations that define this variety. This minimal set of equations, however, is asymmetric causing a different total score depending on the ordering of the pairs within the quartet trees.

Eriksson describes an extended list of 49 invariants which define the Jukes Cantor variety and do not depend on the ordering of the pairs within the quartet trees [10]. We call these *biologically symmetric invariants* or *BSI*. Though longer, we prefer using this list of equations since it makes the selection of a tree with the smallest score independent of the ordering of the taxa. To construct the BSI equations one begins with the list of 33 nonlinear invariants in *q*-coordinates for the Jukes-Cantor model of evolution on the unrooted tree (12)(34) as found on the small trees website [13]. For each of the biologically equivalent tree topologies (ie (12)(43) , (21)(34) , and (21)(43) ) one identifies the corresponding change in *q*-coordinates and then makes the change of coordinates in each of the invariants. This results in a new list of 33 expressions. If any additional invariants appeared, they are added to the list of invariants. After repeating this process for each equivalent tree, there are 14 additional expressions, bringing the total number of biologically symmetric invariants to 49 (including the Lake’s two linear invariants which were already symmetric). When choosing a tree for a quartet (1,2,3,4) we select the tree for which the average of the absolute values of all of the biologically symmetric invariants is the smallest. When these new invariants are evaluated on the example species subset of crab data, we get the scores in Table 2. Using BSI we would select the quartet tree (14)(23).

**Table 2:** Biologically symmetric invariant scores

**Ranking**	**Unrooted Tree**	**Score**
1	(14)(23)	.00595688
2	(13)(24)	.00601559
3	(12)(34)	.00632047

Biologically symmetric scores for a four species subset of crab data.

While it is possible to reconstruct biologically symmetric invariants for the Kimura 2 parameter model as well, we do not include that information here, as reconstruction using these invariants performed significantly worse than those simply using the Kimura 2 parameter invariants.

### Invariant based quartet puzzling algorithm

Our method follows the original quartet puzzling algorithm described in [16]. We begin by using invariants to select the appropriate tree for each of the $\left(\begin{array}{c} n\\ 4 \end{array}\right)$ quartets of data from our sample of *n* taxa. Due to the nature of the invariant scoring, it is highly unlikely that there will be a tie between scores, but in this rare event we randomly select a tree from those with the lowest scores. Next we select *k* orderings of the *n* taxa. Following the recommendations for the tree puzzling algorithm as described in the simulation results of Ranwez and Gascuel [18], we choose *k *= 1,000 random orderings of the taxa unless otherwise specified. For each ordering, we use the BSI to select the best quartet tree for the first four taxa. Additional taxa are added to the tree following the quartet puzzling algorithm [16]. When there is a tie among edges which are candidates for adding the additional taxa, an edge is selected at random from those tied as the most likely edge. For each of the *k* orderings, the algorithm produces an unrooted bifurcating tree with *n* taxa. In the final step of reconstruction, we use the CONSENSE program, which is a part of the PHYLIP software package, to compute an unrooted consensus tree [25]. Our reconstruction program is available at http://faculty.winthrop.edu/hippb/QuartetPuzzlingWithBSI. While there have been many advances in quartet puzzling over the past years (see [17,20,26] for example) we used the traditional puzzling algorithm to allow a more true comparison between invariant based puzzling and traditional maximum likelihood puzzling.

### Simulation study

Since maximum likelihood based quartet puzzling is the most similar model of phylogenetic reconstruction to our model, we tested our model using the data sets of Ranwez and Gascuel [18]. These data sets, which include 6 different tree topologies with 12 taxa, were used to analyze various quartet puzzling based algorithms and improvements. Images of these trees along with corresponding data sets can be found http://www.atgc-montpellier.fr/quartet/. Three of the trees (AA, BB and AB) satisfied the molecular clock assumptions, while three trees (CC, DD and CD) did not. For each tree we have data sets of length 300 and 600 base pairs which were generated using the Seq-gen software [27] under the Kimura two-parameter model with a transition/transversion rate of 2. Each tree and base pair length is run under four different assumptions of evolutionary rate. The exact specifications appear in [18], but can be described in terms of the average maximum pairwise distance (MD). The four data sets consist of low (*MD *≈ 0.1), medium (*MD *≈ 0.3), fast (*MD *≈ 1.0) and very fast (*MD *≈ 2.0) substitution rates per site.

Following [18] we compared the results using the accuracy with which our algorithm reconstructed the correct tree, and the average Robinson-Foulds distance [28] between our reconstructed tree and the actual tree. To compute the Robinson-Foulds distance, one notices first that every internal edge of an unrooted tree partitions the taxa into two disjoint sets known as a split. The Robinson-Foulds distance between two trees counts the number of splits which appear in one tree but not the other. As such, this score ranges from 0, for equivalent trees, to twice the number of internal edges (2(*n*−3) for a unrooted tree with *n* taxa). As the distribution of Robinson-Foulds distances is weighted very heavily to the high end of the score, almost all small scores can be viewed as indicators of at least partial reconstructive success [29]. For the study we analyzed the data sets of Ranwez and Gascuel using Jukes Cantor invariants (JC), biologically symmetric Jukes Cantor Invariants(BSI-JC), the relevant Jukes Cantor invariants (JC-R) and the Kimura 2 parameter invariants (K2P).

To determine the effect of the choice of model selection on phylogenetic accuracy we designed a model misspecification analysis test. We used Seq-gen software [27] to generate 1000 sequences of lengths 300,600 and 5,000 for trees *AA* and *CC* for the four evolutionary rates described above. The sequences were generated using the Jukes Cantor model of evolution, and then separately using the Kimura-2 parameter model of evolution. We reconstructed the trees using BSI Jukes Cantor invariants and Kimura 2 parameter invariants.

## Results

### Comparison with maximum likelihood based quartet puzzling

The results of our simulation study are recorded in the four tables below. Table 3 describes the accuracy of the algorithm in reconstructing the correct tree for length 300 sequences. Table 4 shows the average Robinson-Foulds distance between the correct tree and the reconstructed tree. Tables 5 and 6 list the accuracy and distance results for sequences of length 600 base pairs. We list all data in relation to the results found on the same data set for the quartet puzzling algorithm, listed as QP_4.2_in [18]. Note that the results listed for Ranwez and Gascuel were created using quartet puzzling with a majority-rule consensus method, which does not always lead to a fully resolved tree. Use of this most recent version of the ONSENSE program’s extended majority-rule consensus method would increase the percentage of trees constructed correctly, but also increase the average Robinson-Foulds distance.

**Table 3:** Length 300 simulation accuracy percentages

	**Molecular Clock**				**No Molecular Clock**			
	**AA**	**BB**	**AB**	**AVG**	**CC**	**DD**	**CD**	**AVG**
M=0.1 BSI-JC	5	9	8	7	11	10	12	11
M=0.1 K2P	5	8	6	6	10	10	11	11
M=0.1 ML	1	3	2	2	3	3	4	3
M=0.3 BSI-JC	14	27	17	19	33	34	35	34
M=0.3 K2P	5	22	11	13	27	25	25	26
M=0.3 ML	4	14	7	9	18	24	21	21
M=1.0 BSI-JC	3	11	3	6	20	25	23	23
M=1.0 K2P	1	7	2	3	5	7	6	6
M=1.0 ML	0	3	1	2	17	26	22	22
M=2.0 BSI-JC	0	0	0	0	0	0	0	0
M=2.0 K2P	0	0	0	0	0	0	0	0
M=2.0 ML	0	0	0	0	1	3	1	2

Comparison of the percentage of reconstructed trees that match the correct tree using Biologically Symmetric Invariants (BSI) and Kimura 2 parameter invariants (K2P) versus Maximum Likelihood (ML) for sequences of length 300.

**Table 4:** Length 300 simulation Robinson-Foulds distances

	**Molecular Clock**				**No Molecular Clock**			
	**AA**	**BB**	**AB**	**AVG**	**CC**	**DD**	**CD**	**AVG**
M=0.1 BSI-JC	4.9	4.8	4.9	4.9	4.4	4.5	4.3	4.4
M=0.1 K2P	5.1	4.9	5.0	5.0	4.7	4.7	4.4	4.6
M=0.1 ML	4.3	3.8	4.1	4.1	3.6	3.7	3.6	3.6
M=0.3 BSI-JC	3.2	2.6	3.3	3.0	2.1	2.1	2.1	2.1
M=0.3 K2P	4.4	3.3	4.0	3.9	2.7	2.6	2.7	2.7
M=0.3 ML	3.1	2.2	2.9	2.7	1.8	1.7	1.8	1.8
M=1.0 BSI-JC	4.8	4.8	5.3	5.0	3.1	2.8	2.9	2.9
M=1.0 K2P	5.9	5.7	6.1	5.9	5.9	5.5	5.7	5.7
M=1.0 ML	4.3	3.6	4.1	4.0	1.9	1.7	1.8	1.8
M=2.0 BSI-JC	9.1	11.6	10.3	10.3	11	11.4	11.2	11.2
M=2.0 K2P	8.8	11.7	9.9	10.1	15.1	15.0	14.9	15.0
M=2.0 ML	6.6	6.5	6.6	6.6	4.3	4.1	4.2	4.2

Comparison of the average Robinson-Foulds distance between the reconstructed tree and the correct tree for sequences of length 300.

**Table 5:** Length 600 simulation accuracy percentages

	**Molecular Clock**				**No Molecular Clock**			
	**AA**	**BB**	**AB**	**AVG**	**CC**	**DD**	**CD**	**AVG**
M=0.1 BSI-JC	36	40	37	38	50	45	49	48
M=0.1 K2P	28	35	30	31	47	44	44	45
M=0.1 ML	17	29	21	22	29	28	27	28
M=0.3 BSI-JC	57	66	63	62	77	80	80	79
M=0.3 K2P	33	57	43	44	70	69	69	69
M=0.3 ML	48	61	54	54	74	79	78	77
M=1.0 BSI-JC	23	41	29	31	70	71	68	70
M=1.0 K2P	9	36	16	20	32	36	36	35
M=1.0 ML	21	43	26	30	76	84	79	80
M=2.0 BSI-JC	0	2	0	1	2	5	2	3
M=2.0 K2P	0	4	1	2	0	0	0	0
M=2.0 ML	0	1	0	1	24	36	30	30

Comparison of the percentage of reconstructed trees that match the correct tree using Biologically Symmetric Invariants (BSI) and Kimura 2 parameter invariants (K2P) versus Maximum Likelihood (ML) for sequences of length 600.

**Table 6:** Length 600 simulation Robinson-Foulds distances

	**Molecular Clock**				**No Molecular Clock**			
	**AA**	**BB**	**AB**	**AVG**	**CC**	**DD**	**CD**	**AVG**
M=0.1 BSI-JC	1.9	1.7	1.9	1.8	1.4	1.6	1.5	1.5
M=0.1 K2P	2.4	2.1	2.6	2.3	1.6	1.7	1.7	1.6
M=0.1 ML	1.9	1.5	1.7	1.7	1.4	1.5	1.5	1.5
M=0.3 BSI-JC	1.0	0.8	0.9	0.9	0.5	0.5	0.5	0.5
M=0.3 K2P	1.9	1.2	1.6	1.5	.7	.7	.7	.7
M=0.3 ML	0.8	0.6	0.7	0.7	0.4	0.3	0.3	0.3
M=1.0 BSI-JC	2.3	1.7	2.3	2.1	0.7	0.7	0.8	0.7
M=1.0 K2P	3.6	2.2	3.0	2.9	2.2	2.0	2.0	2.1
M=1.0 ML	1.8	1.0	1.5	1.4	0.4	0.3	0.3	0.3
M=2.0 BSI-JC	6.7	7.8	7.6	7.4	7.4	6.9	7.2	7.2
M=2.0 K2P	6.3	7.0	6.8	6.7	11.6	11.8	11.8	11.7
M=2.0 ML	4.4	3.8	4.3	4.2	1.7	1.4	1.6	1.6

Comparison of the average Robinson-Foulds distance between the reconstructed tree and the correct tree for sequences of length 600.

### Variations of invariant based quartet puzzling

To compare the difference between using the entire list of Jukes Cantor biologically symmetric invariants (BSI-JC) to only the relevant invariants (JC-R) we ran the simulation study again using only the relevant invariants. The average accuracy for the blocks of datasets are listed in Table 7. On these same data sets we compared the accuracy of quartet puzzling with BSI invariants to those using the minimal invariants. Table 8 lists the comparison of these methods in terms of accuracy. The choice of type of invariants used in the puzzling algorithms had a negligible effect on the computation time.

**Table 7:** Comparison of BSI with relevant invariants only

	**L=300 Mol. clock**	**L=600 Mol. clock**	**L=300 No clock**	**L=600 No clock**
M=0.1 BSI-JC	7	38	11	48
M=0.1 JC-R	10	41	11	45
M=0.3 BSI-JC	19	62	34	79
M=0.3 JC-R	24	69	32	78
M=1.0 BSI-JC	6	31	23	70
M=1.0 JC-R	5	26	14	48
M=2.0 BSI-JC	0	1	0	3
M=2.0 R-JC	0	1	0	2

Comparison of percentage of reconstructed trees that match the correct tree using biologically symmetric invariants (BSI-JC) compared with using only the relevant invariants (JC-R).

**Table 8:** Comparison of BSI-invariants to minimal invariants

**Tree**	**BSI-JC**	**JC**
CC M=0.1	11	10
CC M=0.3	33	32
CC M=1.0	20	15
CC M=2.0	0	0
CD M=0.1	12	10
CD M=0.3	36	32
CD M=1.0	21	17
CD M=2.0	0	0
DD M=0.1	10	9
DD M=0.3	34	32
DD M=1.0	23	20
DD M=2.0	0	0

Percentage of reconstructed trees that match the correct tree using BSI invariants (BSI-JC) vs minimal Jukes Cantor invariants (JC) (length=300 bp).

To compare our method with traditional quartet puzzling, we ran the program with *k *= 1000 random orderings of the data. The number of orderings was chosen to match the conditions of the study in [18]. We also tested the effect of using a smaller number of orderings on both the speed and reconstruction accuracy. The accuracy results are described in Table 9. We ran our program on a Dell Optiplex 960 with an Intel 2 Duo 3 GHz processor and 3.5 GB of RAM. The average run time per tree for *k *= 1000 orderings was 1.9 seconds. For *k *= 100 orderings the run time was 0.3 seconds, and with *k *= 10 orderings the run time was 0.1 seconds per tree.

**Table 9:** BSI-JC accuracy based on number of orderings

**Tree**	**k=1000**	**k=500**	**k=100**	**k=50**	**k=10**
CC M=0.1	11	10	11	11	10
CC M=0.3	33	33	34	32	27
CC M=1.0	20	19	18	18	14
CC M=2.0	0	0	0	0	0
CD M=0.1	12	13	13	12	11
CD M=0.3	36	33	34	33	27
CD M=1.0	21	22	20	19	14
CD M=2.0	0	0	0	0	0
DD M=0.1	10	10	10	10	9
DD M=0.3	34	34	34	33	28
DD M=1.0	23	24	22	22	18
DD M=2.0	0	0	0	0	0

Percentage of reconstructed trees that match the correct tree using N randomly generated orderings (length=300 bp).

### Model selection analysis

The results of our analysis on the effect of model selection on invariant based quartet puzzling are described in Tables 10 and 11. Table 10 summarizes the average effect of using BSI-JC invariants versus K2P invariants for data sets generated from trees AA and CC of length 300 and 600 for low, medium and high evolutionary rates. With 1000 sequences generated for each type for a total of 12,000 sequences included in the average. For length 300 or 600 sequences neither algorithm reconstructed a significant number of the trees accurately. For length 5,000 trees, each model reconstructed the correct tree with near 100 percent accuracy with the exception of trees generated with a very high evolutionary rate (*MD*≈2.0). Table 11 summarizes the results for these longer sequences.

**Table 10:** Effect of model selection on reconstruction accuracy for sequences of length 300 and 600 with low, medium or high evolutionary rates

	**Jukes Cantor BSI Invariants**	**Kimura 2 Parameter Invariants**
Sequences generated under Jukes Cantor model assumptions	35.3	31.3
Sequences generated 2 under Kimura Parameter model assumptions	34.1	23.0

Percentage of reconstructed trees for data data sets generated by Jukes Cantor versus Kimura 2 parameter model assumptions.

**Table 11:** Effect of model selection on reconstruction accuracy for sequences of length 5000 with very high evolutionary rates

	**Jukes Cantor BSI Invariants**	**Kimura 2 Parameter Invariants**
Sequences generated under Jukes Cantor model assumptions for tree AA	24	12
Sequences generated under Kimura 2 Parameter model assumptions for tree AA	56	69
Sequences generated under Jukes Cantor model assumptions for tree CC	99	84
Sequences generated under Kimura 2 Parameter model assumptions for tree CC	63	88

Percentage of reconstructed trees for data data sets generated by Jukes Cantor versus Kimura 2 parameter model assumptions.

## Discussion

### Simulation study of BSI Jukes Cantor and Kimura 2 parameter quartet puzzling

Based on our simulated data sets, BSI-JC quartet puzzling more frequently reconstructs the correct evolutionary tree for trees constructed with low to medium evolutionary rates in comparison with ML-Quartet Puzzling. For length 300 sequences with low to high rates of evolution (*MD *≤ 1.0 ), BSI- quartet puzzling was twice as likely as ML-Quartet Puzzling to reconstruct the exact twelve taxa tree (18% vs. 9%). For length 600 sequences with the same rates of evolution BSI-quartet puzzling provides a modest improvement in exact reconstruction accuracy as well (57% vs 45%). For very high rates of evolution, both methods do poorly at reconstructing the exact tree (≤ 2*%*) with the exception of ML reconstruction in length 600 trees without a molecular clock (30*%*) .

Even in instances where BSI-JC outperforms ML in reconstruction accuracy, the average Robinson-Foulds distance [28] remains slightly larger. This indicates that while BSI-JC quartet puzzling is more likely to reconstruct the exact correct tree, the number of splits which are correctly reconstructed would be smaller. Our results indicate that this error is typically on the order of one additional misconstructed split for every four reconstructions if the evolutionary rate is high, medium, or small (M ≤ 1.0 ), and an additional two and a half misconstructed splits per reconstruction if the evolutionary rate is high (*DL *= 2.0) .

Given that the data sets used to run this simulation study were generated under the Kimura 2 parameter model assumptions it is quite surprising that the K2P invariants under perform the BSI-JC model for almost all trees. The only region where K2P invariants outperformed JC-BSI was for trees satisfying a molecular clock assumption and a very high rate of evolution. We believe the under-performance of the K2P invariants may be attributed to additional noise in the data created by the large number of invariant expressions. Recent work has indicated that certain individual invariants may be biased against reconstructing the correct quartet tree [10]. We believe a more nuanced scoring system could account for this bias and improve the accuracy of reconstruction algorithms using BSI-JC and K2P invariants.

### Variations of invariant based quartet puzzling

The transition from the Jukes Cantor invariants (JC) on the small trees website [13] to biologically symmetric invariants (BSI-JC) has a minor effect on improving reconstruction accuracy. The extra time required to evaluate the additional biologically symmetric invariants is made up for by slightly improved accuracy and dramatically improved peace of mind.

Restricting the equations to use only the relevant invariants had limited success. While mild accuracy improvements were seen at low to medium rates of evolution, there were significant loses of accuracy on trees simulated without a molecular clock and with a high evolutionary rate. This suggests that the cubic invariants, while not relevant may still play an important role in reconstructing certain trees. Our data confirms that the relevant invariants are a sufficient set of equations for performing reasonable phylogenetic analysis [21].

All comparisons between BSI and ML based quartet puzzling were made using consensus trees based on 1000 random taxa orderings. This number was selected to match the number of runs used in the puzzling in the simulation study under comparison. Our testing indicates that dramatic increases in speed could be gained with little to no effect on accuracy by running only 50 data orderings. We assume that the 1000 orderings suggestion in the TREEPUZZLE guidelines is there for working with larger data sets and that similar savings in time would occur when running traditional puzzling methods on this data set with fewer orderings. Given the tremendous increase in speed, further investigation into the appropriate number of orderings which maximize a combination of speed and accuracy may be of merit.

### Model selection analysis

The results of the effect of model selection on invariant based reconstruction are surprising. For data sets of length 300 or 600 reconstructions using the Jukes Cantor BSI invariants outperform those using the Kimura 2 parameter invariants regardless of which model was used to create the data sets. This suggests that the Kimura 2 parameter invariants are not being utilized in an optimal matter. For length 5,000 sequences with a very high evolutionary rate, trees are more accurately reconstructed using the invariants corresponding to the model used to generate the data. This suggests that when there are many site substitutions in the data, it is important to use the invariants from the appropriate model.

## Conclusion

Given that ML models are known to reconstruct the correct quartet trees with very high accuracy, the fact that BSI-puzzling performs comparably with ML-puzzling in most circumstances is somewhat surprising and encouraging. We see this as evidence that invariant based models of reconstruction may play an important role in practical phylogenetic reconstruction in addition to the role they currently play in helping to understand the theoretical possibilities of phylogenetic reconstruction problems [14].

As incorrect quartet propagation will still occur in the puzzling procedure, we would not expect this method to compete with the many variations of ML based quartet puzzling algorithms that have been developed over the years. Our findings, should be viewed as a proof of principle that invariant based algorithms for phylogenetic reconstruction are practical, and should be of interest to working biologists, not just phylogenetic algorithm specialists.

The issue of using the full list of invariants as opposed to only the *relevant* invariants remains unresolved. For trees with a molecular clock assumption and small rates of evolution, the relevant invariants outperformed the complete list. However, for non-molecular clock trees, and molecular clock trees with higher rates of evolution, the complete list of invariants outperformed the relevant invariants. We believe further study is needed in this area. While we agree in principle that a smaller list of invariants would be beneficial, our data suggests that the cubic invariants do play an important role in reconstructing trees.

In this paper we do not claim to have fully exploited the power of invariants to solve issues such as long branch attraction. We believe that a more nuanced large scale investigation of invariant based algorithms may provide solutions to traditional problems in phylogenetic reconstruction. As genetic data is becoming more readily available, and as scientists seek to assemble the Tree of Life, the importance of accurate and fast phylogenetic analysis is extremely important. Our findings indicate that invariant based algorithms should be included in the search for these improved algorithms, especially when computing the Tree of Life, where current algorithms focus around quartet based methods of reconstruction [30].

## Competing interests

The authors declare that they have no competing interests.

## Authors’ contributions

JR designed the algorithm and simulation studies. BH designed and tested the software. Both authors drafted the manuscript. Both authors read and approved the final manuscript.
